# Response of streamflow and nutrient loads in a small temperate catchment subject to land use change

**DOI:** 10.1007/s10661-023-11828-z

**Published:** 2023-11-06

**Authors:** Gebiaw T. Ayele, Bofu Yu, Andy Bruere, David P. Hamilton

**Affiliations:** 1 Australian Rivers Institute and School of Engineering, Griffith University, Nathan, Queensland 4111 Australia; 2 Bay of Plenty Regional Council, Rotorua, New Zealand

**Keywords:** Land use change, Hydrological modelling, Statistical test, SWAT, Non-stationarity, Water quality, Lake Ōkareka, New Zealand

## Abstract

The aim of this study was to quantify the effect of land use change (LUC) implemented to meet nutrient load targets for a freshwater lake in New Zealand. We used the Soil and Water Assessment Tool (SWAT) model in combination with a non-parametric statistical test to determine whether afforestation of 15% of a subcatchment area was adequate to meet assigned nutrient load targets. A regional management authority set nutrient load targets of reduction in total nitrogen (TN) by 0.9 t yr^−1^ and reduction in total phosphorus (TP) by 0.05 t yr^−1^ to avoid eutrophication in the receiving waters of a freshwater lake. The load reduction was designed to be achieved through 200 ha of LUC from pasture to trees. Analysis of nutrient loads before, during, and following LUC shows that a 15% increase in forest cover decreased the annual flow (7.2%), TP load (33.3%), and TN load (13.1%). As flow and water quality observations were discrete and at irregular intervals, we used a parametric test and the SWAT model as different lines of evidence to demonstrate the effect of afforestation on flow and water quality. Policymakers concerned with decisions about LUC to improve the quality of receiving waters can benefit from applying our findings and using a statistical and numerical modelling framework to evaluate the adequacy of land use change to support improvements in water quality.

**Supplementary Information:**

The online version contains supplementary material available at 10.1007/s10661-023-11828-z.

## Introduction

Increased human population and consumption patterns have created global demand for agricultural products, which has often adversely affected ecosystem services and biodiversity (MEA, 2005; Nelson et al., 2010). Landscape disturbance associated with agricultural production leads to increased mobilisation of sediment and nutrients, with many mitigation actions designed to minimise or offset the effects of disturbance and avoid impaired water quality (Mello et al., 2020). These actions often include land use change (LUC), many involving afforestation, and altered management practices. An understanding of the hydrological connectivity between areas of sediment and nutrient mobilisation and receiving waters within catchments is important to predict the impacts of LUC and other management practices on receiving water environments (Leibowitz et al., 2018).

Hydrological models are valuable tools to quantify discharge and nutrient loads that affect the water quality and ecological status of receiving waters and to assess temporal changes associated with LUC (Milly et al., 2008). Ability to capture these features can help identify nutrient ‘hotspots’ and ‘hot moments’ (Harms & Grimm, 2008) and support informed policy decisions about prioritising areas within catchments and sub-catchments for intensive management and LUC. Confidence in assessments of LUC impacts on flow and nutrient loads is often based on demonstrating ‘good’ fit of the model output to observations, using statistical metrics for calibration and validation periods (Chen et al., 2019). However, one of the challenges in modelling catchment processes can be a paucity of high-quality data. For ‘data-poor’ catchments, non-parametric tests provide a useful statistical tool to compensate for the effects of limited data, particularly when data are non-normally distributed (Mohebbi & Akbariyeh, 2021) or there are missing data (Naddeo et al., 2013). Non-parametric percentile estimation techniques can complement conventional methods that rely on statistical assessment of model calibration and validation periods to describe the distribution of flow and changes in water quality variables.

Declines in water quality have been widely attributed to the effects of agricultural development, deforestation, urbanisation, and land use change. Distributed hydrological models, which include input parameters in relation to land use, have been applied to assess LUC impacts on runoff and changes in nutrient loads (El-Khoury et al., 2015). The Soil and Water Assessment Tool (SWAT; Arnold et al., 1995) has been used to predict the quality and quantity of water and assess the environmental impacts of LUC. SWAT accounts for spatial heterogeneity of land use and the effectiveness of erosion control measures (Arnold et al., 1995) as well as land use change by updating an initial prescribed LUC distribution through the simulation period (Moriasi et al., 2019). SWAT is the most widely used hydrological model globally (Tan et al., 2020) and is supported by an online literature database of over 4500 articles from 1993 to 2023 (e.g. www.card.iastate.edu/swat_articles/). A review of SWAT model applications (Gassman et al., 2007) citing more than 250 journal articles indicated that model performance was mostly adequate to make assessments about LUC and non-point source (NPS) pollution and land use-climate change impact (Douglas-Mankin et al., 2010).

Khoi et al. (2022) used the SWAT model to assess water quantity and quality (e.g. sediment, total nitrogen, and total phosphorus) responses to changes in different land use types; agricultural, bush, forestland, and urban areas. This study highlighted an inverse relationship between forest cover and water quantity. The SWAT model has also been used to assess LUC effects on water quantity as a result conversion of forest to oil palm plantation (Tan et al., 2015). Other studies (Hoghooghi et al., 2021) have used SWAT to assess the effect of *Brassica carinata* plantations on flow, sediment, and nutrients loads in an experimental watershed in the upper Suwannee River Basin, South-Central Georgia. Plantations covering 12% of the watershed resulted in reductions in total sediment, mineral phosphorus, and nitrate loads ranging from 3.8 to 14.0%. A study using SWAT in the Big Sunflower River Watershed in the Mississippi Delta, USA, assessed the effect of spatiotemporal changes in land use classes, such as forest, cropland, corn, and soybean (Ni et al., 2021), while Nguyen et al. (2019) used SWAT to generate scenarios of flow and pollutant loads arising from land use intensification.

Agriculture has intensified in New Zealand over recent decades, resulting in increased NPS nutrient discharge that has impaired the water quality of streams (McDowell et al., 2014; Snelder et al., 2018) and lakes (Abell et al., 2020a, b). Declining water quality ranks as the foremost environmental concern in surveys of the New Zealand population (Hughey et al., 2019). In response to these concerns, the New Zealand government has implemented a National Policy Statement for Freshwater Management (NPS-FM) (Ministry for the Environment, 2020). The NPS-FM directs regional and local councils to set limits for concentrations of sediment, phosphorus, nitrogen, and microbial contaminants in waterbodies (Journeaux et al., 2017). Changes in land use or land management are the primary levers used by councils to achieve compliance with the NPS-FM (McDowell et al., 2014; Snelder et al., 2018). For example, for New Zealand’s largest lake, Taupō (area = 616 km^2^; Central Volcanic Plateau, North Island), market-based environmental policies involving nitrogen trading have been established (Duhon et al., 2015), linked to LUC from agriculture to forest to reduce NPS loads. In other areas, regional plans and policies include directives for catchment management to meet nitrogen and phosphorus load targets for lakes (Abell et al., 2011), many involving afforestation. However, implementing interventions can be challenging due to a diversity of land uses, active LUC, and climate variability in New Zealand (Me et al., 2018), as well as difficulty in change detection (e.g. for nutrient loads).

To implement effective management policies to reduce the impacts of nutrients, assessments are required to identify the relative contributions of non-point and point sources of nutrients (Abell et al., 2020a, b; Roygard et al., 2012). Elliott et al. (2005) used the SPAtially Referenced Regression on Watershed (SPARROW) model and the National (New Zealand) River Water Quality Network dataset to indicate that non-point loads exceed 96% of the total nutrient load across New Zealand; NPS pollution is therefore one of the most challenging environmental problems for New Zealand (Snelder et al., 2018).

Our study was designed for sub-catchments where flow and water quality data are typically collected infrequently; a situation common to many other catchments where LUC (afforestation) is being implemented to meet water quality targets. The study focused on a lake catchment in New Zealand where there were infrequent discrete observations of flow and water quality, which is typical of many catchments globally. The regional council action plan for the lake indicated that 200 ha LUC from pasture to trees would be adequate to avoid eutrophication of the downstream receiving waters of Lake Ōkareka. Building upon findings from prior research (Farley et al., 2005; Vertessy, 2001; Zhang et al., 2001; Zhang et al., 2003), our hypothesis was centred around the notion that there would be a significant difference in the average streamflow and nutrient loads between pre- and post-LUC values. The hypothesis is consistent with the concept that when an area undergoes afforestation, the dense canopy and extensive root network of trees and vegetation increase rainwater interception, reducing surface runoff and subsequent streamflow, as well as supporting nutrient retention. We tested the extent of LUC required to meet the nutrient load targets for the lake. The objectives of this study were to (1) test for significant changes in flow and nutrients measured pre- and post-LUC, (2) implement a non-parametric percentile estimation technique to examine shifts in the frequency domain between observations and model output, (3) assess the ability of the SWAT model to reproduce observed streamflow and nutrient loads arising from LUC, and (4) assess the resulting changes in seasonal variability of water quantity and quality. First, we analysed a time series of observations of responses of flow and nutrients to LUC at the sub-catchment scale within the Lake Ōkareka catchment. Next, we simulated streamflow and catchment nutrient export in a sub-catchment of the lake using SWAT. Last, we combined results from non-parametric tests and the SWAT model to describe the changes in flow and water quality variables.

## Data and methods

### Study area

**Fig. 1:**
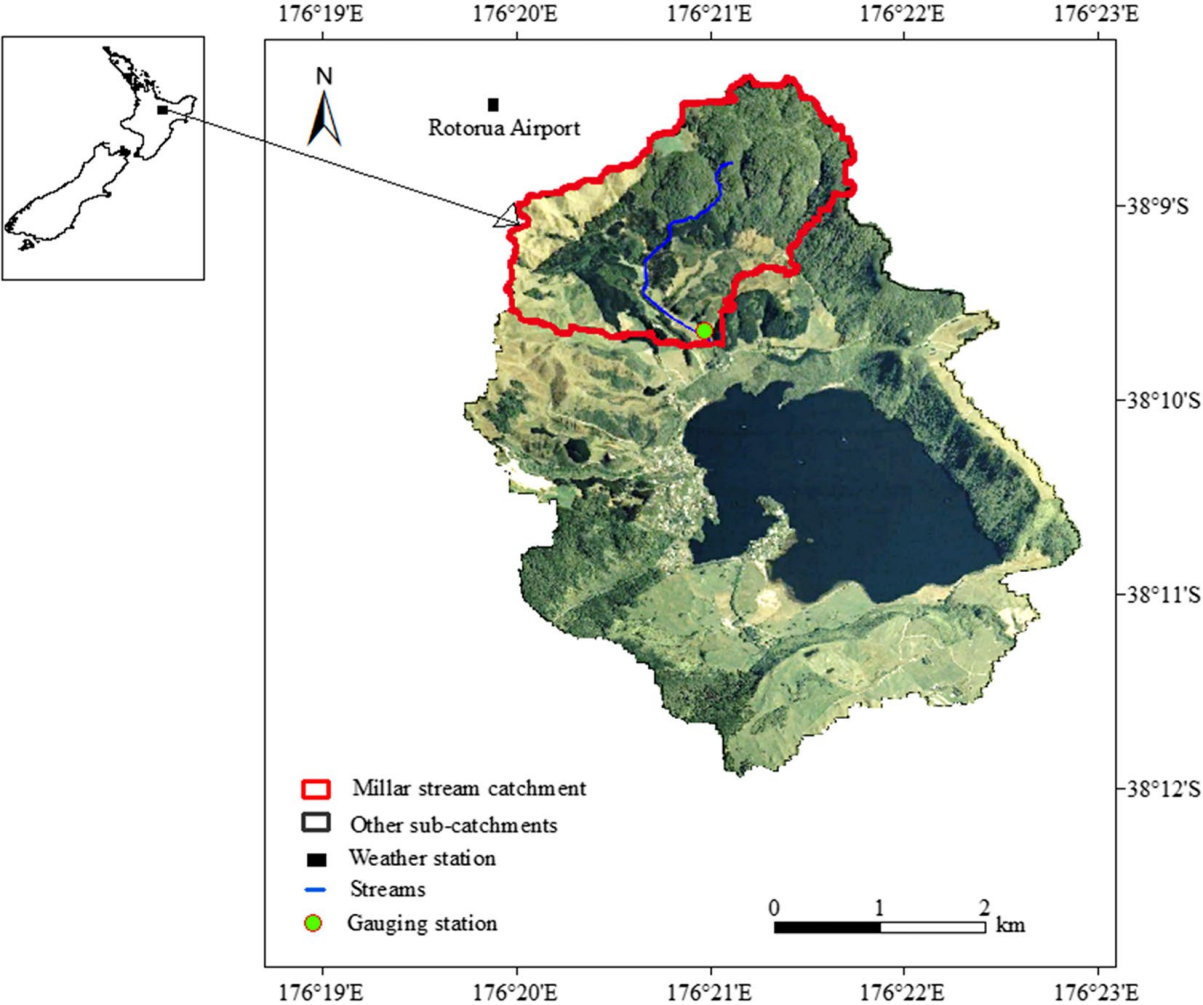
Location map of the study area including Millar sub-catchment in the Lake Ōkareka catchment and the gauging station. Inset: Map of New Zealand showing location of Lake Ōkareka

The Ōkareka catchment (Fig. 1) is located in the Rotorua District of the North Island of New Zealand. The geology of the area is characterised by volcanic and sedimentary deposits (Healy, 1964) with a series of rhyolitic domes and lava flows flanking the west, south, and east of the lake (Nairn, 2002). The catchment area is 1500 ha and drains to monomictic Lake Ōkareka. The lake is important recreationally and culturally. Elevations in the catchment range from 335 to 685 m above mean sea level. The mean annual precipitation in the area is about 1300 mm, with considerable interannual variation. Annual mean air temperature, relative humidity, and wind speed measured 15 km from the lake at Rotorua airport are 13.4 °C, 82%, and 3.6 m s^−1^, respectively (National Climate Database; cliflo.niwa.co.nz/). The modelling study was conducted on the Millar sub-catchment (area = 384 ha) because of the availability of relatively long-term monitoring data (Fig. 1). Millar stream is the main surface water inflow to the lake and this sub-catchment covers 26% of the total catchment area (Fig. 1). Summary of the land use information in the Millar stream sub-catchment indicates that compared with a 2003 baseline, 17 ha (4.4% of Millar sub-catchment area) had been planted in pine (*Pinus radiata*) or native trees by 2011, 53 ha (13.8%) by 2013, and 57 ha (15%) by 2015. Recent studies of the lake have classified its trophic state as mesotrophic (Trolle et al., 2010) after it was previously assessed to be oligotrophic (McColl, 1972). Mesotrophic refers to a water body with moderate nutrient levels, supporting balanced plant growth and diverse aquatic life, and oligotrophic denotes waters with low nutrient levels, resulting in clear, pristine conditions and limited biological productivity (Burns et al., 2009). Millar sub-catchment has moderately steep topography, and pasture and native forest cover typified by a climate of cold winters and mild to cool summers. After 2010, there has been significant afforestation including *Pinus radiata* and native forest.

### Testing for temporal changes in observed flow and water quality variables

We used parametric, cumulative deviation (CDT), and non-parametric percentile estimation to describe distributions of streamflow and nutrient loads as we had discrete and instantaneous observations of discharge, sediment, and nutrient concentrations (Mohebbi & Akbariyeh, 2021; Naddeo et al., 2013). We split data based on the proportion of observations below and above a given value in a selected percentile distribution (McCluskey & Lalkhen, 2007). Flow and loads of ammonium (NH_4_-N), nitrate (NO_3_-N), total nitrogen (TN), and total phosphorus (TP) for each monitoring day, and 5-day total rainfall, normalised by the respective maximum values were examined pre- and post-LUC. In addition, we used descriptive statistics such as the mean, standard deviation (SD), minimum, and maximum values to compare Q, NH_4_-N, NO_3_-N, TP, and TN for pre- and post-LUC periods.

Cumulative deviation was used to detect temporal trends in the variables Q, NH_4_-N, NO_3_-N, TP, and TN for pre-LUC (2002–2010) and post-2010 periods up to 2021 (Chiew & Siriwardena, 2005). A 5-day mean rainfall was also included as a surrogate for the effect of antecedent soil moisture conditions. CDT detects change in the means for a time period (e.g. related to pre- and post-LUC).

Tests for homogeneity of observations were based on the adjusted partial sum ($\text{S}^{*}_\text{k}$), which is the cumulative deviation from the mean (*ӯ*) for *n* observations of a series *y*_*1*_*, y*_*2*_*, y*_*3,…*_ *y*_*n*_:

1 $${\textrm{S}}_{\textrm{k}}^{\ast }={\sum}_{\textrm{i}}^{\textrm{k}}\left({y}_i-\overline{y}\right),k=1,\dots n$$

The rescaled adjusted partial sum, $\text{S}_\text{k}^{\ast \ast },$ was calculated by dividing the adjusted partial sum ($\text{S}_\text{k}^{\ast }$) by the standard deviation, D_y_:

2 $${\textrm{S}}_{\textrm{k}}^{\ast \ast }={\textrm{S}}_{\textrm{k}}^{\ast }/{\textrm{D}}_{\textrm{y}},k=1,\dots n$$

3 $${\textrm{D}}_{\textrm{y}}^2=\sum\nolimits_{\textrm{i}=1}^{\textrm{n}}{\left({y}_i-\overline{\textrm{y}}\right)}^2/\textrm{n}$$

Departures from homogeneity are defined by *Ơ*, the maximum ${S}_k^{\ast \ast }$ value in the data series. A critical value of a variable is defined by the ratio of *Ơ* to √*n* (Buishand, 1982) where *n* is the number of observations for NH_4_-N, NO_3_-N, TN, TP, or 5-day mean rainfall.

### Soil and Water Assessment Tool (SWAT) model

SWAT is a semi-distributed catchment model that requires static geospatial information (terrain topography, soil, and land use) and dynamic (meteorological) data. Using a digital elevation map (DEM), the model delineates a catchment into multiple sub-catchments, which may be split further into homogeneous Hydrologic Response Units (HRUs) with unique combinations of land use, soil, and slope (Neitsch et al., 2011). Nutrient and sediment transformations and losses are modelled separately for each HRU, with predictions accumulated to obtain the total for each sub-catchment and then routed to the associated reach and catchment outlet through the channel network (Neitsch et al., 2011). Daily observed precipitation, temperature, wind speed, solar radiation, and relative humidity are used as hydrological data to run the model in a daily time step (Neitsch et al., 2011). The climate data is from 2002 to 2021.

SWAT contains a nutrient routing module that simulates total nitrogen (TN) and total phosphorus (TP) concentration (Gassman et al., 2007), including constituents of organic nitrogen (ORGN), ammonium (NH_4_–N), and nitrate (NO_3_–N) (Gassman et al., 2007). TP is defined as the sum of organic phosphorus (ORGP) and mineral phosphorus (MINP) with these fractions commonly taken to be the particulate and dissolved phases of phosphorus (Me et al., 2018).

### SWAT inputs

**Fig. 2:**
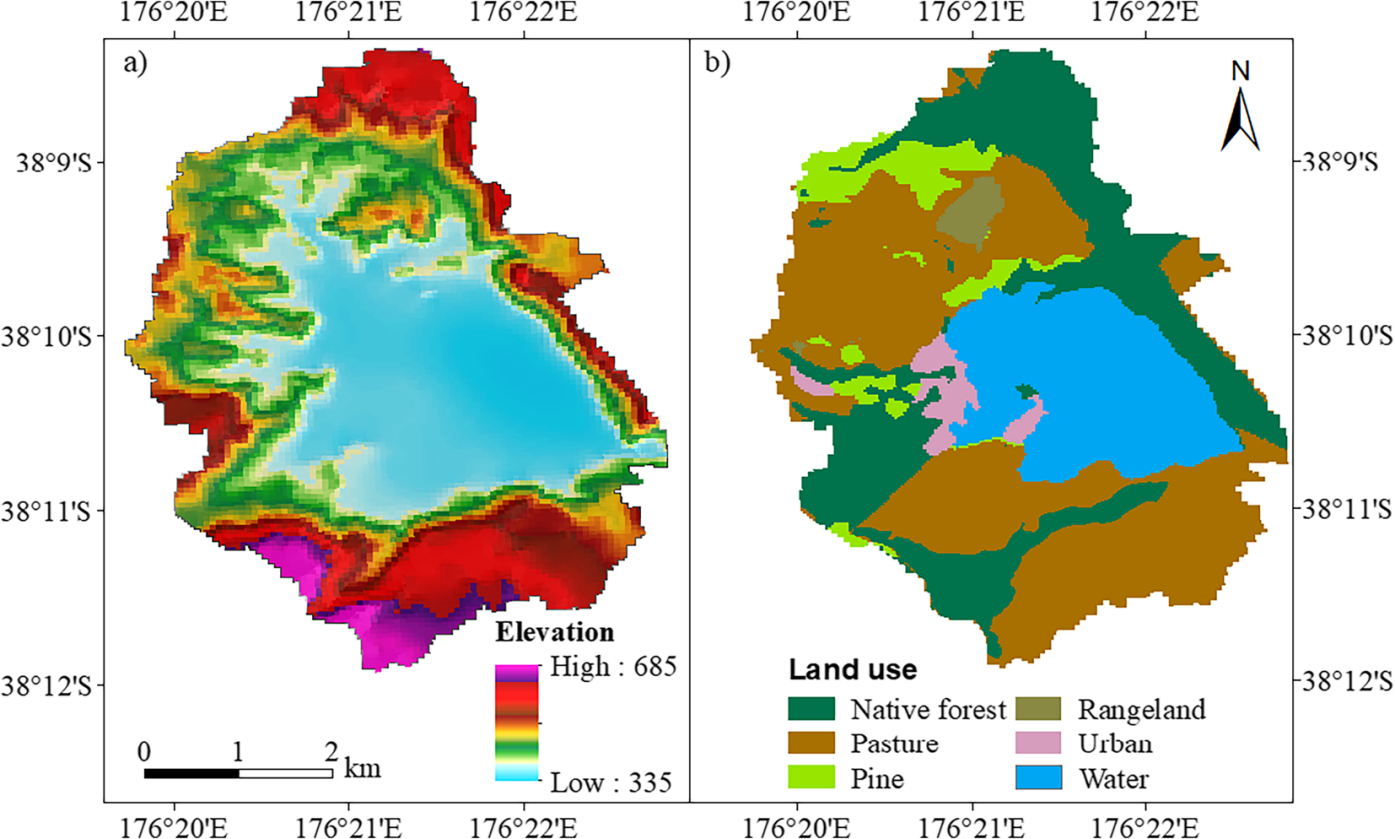
Digital elevation (**a**) and land use (**b**) in the Lake Ōkareka catchment

A digital elevation model with 25-m horizontal resolution was used to generate the stream network and sub-catchments of Lake Ōkareka. Digital data on soil type and physicochemical properties of the area were obtained from Manaaki Whenua New Zealand Land Resource Inventory (NZLRI) and Digital Soil Map (S–map; smap.landcareresearch.co.nz/home). Land use maps were sourced from the New Zealand Land Cover Database v2 (LCDB-2) that used Landsat 7 (Enhanced Thematic Mapper) satellite imagery (www.lcdb.scinfo.org.nz/about-lcdb)*.* Land use and soil data were available at 25-m resolution (Table 1, Fig. 2). Land use in the Ōkareka catchment is a mix of forest (native, pine), pasture, rangeland, medium density residential area, and water bodies (Lake Ōkareka).

**Table 1:** Data description and source to configure the SWAT model for Lake Ōkareka. NH_4_-N, NO_3_-N, TN, and TP are ammonium, nitrate, total nitrogen, and total phosphorus, respectively

Data	Application	Description	Source
Meteorological	Meteorological forcing	Daily max and min temperature, humidity, solar radiation, wind speed, and precipitation	Rotorua Airport Automatic Weather Station, National Climate Database (available at: cliflo.niwa.co.nz/)
DEM and digitised stream network	Catchment delineation	25-m resolution to define slope classes	Bay of Plenty Regional Council (BoPRC)
Land use	HRU definition	25-m resolution, 6 basic land-cover classes	High temporal resolution Landsat 7 satellite imagery supporting LCDB-2
Soil characteristics	HRU definition	25-m resolution, 9 soil types	NZLRI and Digital Soil Map (smap.landcareresearch.co.nz/)
Stream discharge measurements	Calibration (2002–2005) Validation (2006–2010)	Instantaneous stream discharge data (2002–2021) were measured at Millar Road stream gauge station (Fig. 1)	BoPRC
Stream water quality measurements	Calibration (2002–2005) Validation (2006–2010)	Instantaneous measurements for determination of NH_4_-N, NO_3_-N, TN, and TP concentrations (2002–2021)	BoPRC

Daily rainfall, temperature, solar radiation, relative humidity, and wind speed model inputs were obtained from Rotorua Airport Automatic Weather Station (The National Climatic Database [New Zealand]; cliflo.niwa.co.nz/). Observed streamflow and concentrations of NH_4_-N, NO_3_-N, TN, and TP were measured instantaneously from Millar Road stream gauge station (Fig. 1) and collected by the Bay of Plenty Regional Council (BoPRC). Most of the measurements were collected monthly, which partly reflects the paucity of comprehensive flow and water quality data. The Millar Road stream gauge station had only limited sediment concentration data, which was deemed not adequate to support model calibration and validation sediment concentration. Detailed information on model input and data sources is presented in Table 1.

Changes in land use have been implemented in the Ōkareka catchment to meet nutrient load targets for the lake (Burns et al., 2009). We inferred spatiotemporal changes in land use with Google Earth Engine based on Landsat 7 Enhanced Thematic Mapper from 2003 to 2013 and Landsat 8 Operational Land Imager from February 2013. Images for years 2003, 2011, 2013, and 2015 were verified with BoPRC observations, focusing on identification of areas of afforestation on pastoral land. Water quality and quantity responses to land use change were accounted for through the land use update (LUP) routine in the SWAT model (Moriasi et al., 2019). LUP sets a land use change update at user-specified day.

### SWAT performance assessment

A standalone SWAT Calibration and Uncertainty Program (Arnold et al., 2012) was used to optimise SWAT model parameters. Selection and configuration of parameters for streamflow and water quality followed the recommendations of Abbaspour et al. (2015). We chose the SUFI-2 routine in the SWAT-CUP program to perform a global sensitivity analysis for identification of sensitive SWAT model parameters (Abbaspour et al., 2004). This routine is computationally efficient in limiting the number of iterations to quantify the effect on model outputs of changes in an input parameter (Yang et al., 2008). Our choice of the range of flow and nutrient-related parameter ranges was consistent with previous studies in the wider region (Me et al., 2018). After identification of model parameter ranges, sensitive parameters, and an autocalibration procedure in SWAT-CUP, we checked and fine-tuned model parameter values using manual adjustment of parameters for a calibration period of 2002–2005, with 2000 to 2001 used as a model ‘warmup’ period. Streamflow was calibrated first, followed sequentially for concentrations of TP, TN, NO_3_-N, and NH_4_-N, and several iterations of this procedure were used. Observed streamflow and concentration data were collected for each monitoring day in a single event and sporadic in nature. The observed flow and water quality data (NH_4_-N, NO_3_-N, TN, and TP) were partitioned into pre- and post-LUC categories and assigned for calibration, validation, and land use change impact analyses. Data were of unequal length with three distinct phases of pre-LUC calibration (2002 to 2005) and validation (2006 to 2010) and post-LUC (post-2010).

In our model setup, calibration and validation were based on measurements of flow and nutrient concentration during pre-afforestation period (2002–2010) as there was no visible land use change in the catchment. Since reforestation, as a land use change, occurs gradually over time rather than instantaneously, calibrating the model during this period could lead to uncertainties and errors, as the system is in a state of transition. On the other hand, choosing data sets prior to land use change provides a more stable and consistent baseline for model calibration, helping to avoid overfitting and providing more confidence in the model predictions. In addition, our choice of pre-afforestation data (from 2002 to 2010) for model training is related to several other reasons. First, we set the model in view of future LUC scenario development and assess the versatility of the model under dynamic catchment and climatic factors; next, according to Moriasi et al. (2019), SWAT model has a well-established and considerably versatile system that represents transient LUC in the modelling environment. Therefore, SWAT calibrated and validated with pre reforestation data could be used to predict post reforestation streamflow and water quality response; finally, it appears that the region receives high proportion of flow from base flow stores (Me et al., 2015) and has only moderate seasonality in both pre- and post-afforestation periods.

We used coefficient of determination (*R*^2^), Nash-Sutcliffe Efficiency (NSE), and percent bias (PBIAS) as statistical evaluation criteria for calibration and validation (Moriasi et al., 2007). Equations for each of these measures are given in Table S1. NSE (range: − ∞ to 1.0) gives the relative magnitude of the residual variance compared to the variance in the measured data (Nash & Sutcliffe, 1970). PBIAS describes the tendency of simulated output data to be consistently smaller or larger than the observed data in the mean with values < 25% indicating satisfactory model performance. Moriasi et al. (2019) distinguished model performance ratings of very good, good, satisfactory, and unsatisfactory based on values of these metrics (see Table S1).

### Flow-weighted mean concentrations

Flow-weighted mean nutrient concentration was used to reduce the bias from sparse, discrete observations for the Millar sub-catchment (Meals et al., 2013). We used a nondimensional sensitivity analysis (Chiew, 2006; Sankarasubramanian et al., 2001) to assess the sensitivity of flow to changes in rainfall (i.e. ratio of relative change in streamflow to relative change in rainfall) and sensitivity of nutrient loading to variations in flow. The elasticity of each nutrient variable was quantified from the ratio of relative changes in nutrient load to flow.

Temporal changes in afforestation on streamflow and nutrient loads were examined using the percentage relative change in each variable between a baseline simulation of 20 years (2002–2021) considering no LUC (*S*_0_) and one that included the existing LUC post 2010 (*S*_LUC_):

4 $$\%\textrm{relative}\ \textrm{change}\kern0.5em =\left(^{{S}_{LUC}}/_{{S}_0}-1\right)\ \textrm{x}\ 100$$

## Results

### Observations of changes pre- and post-land use change

**Fig. 3:**
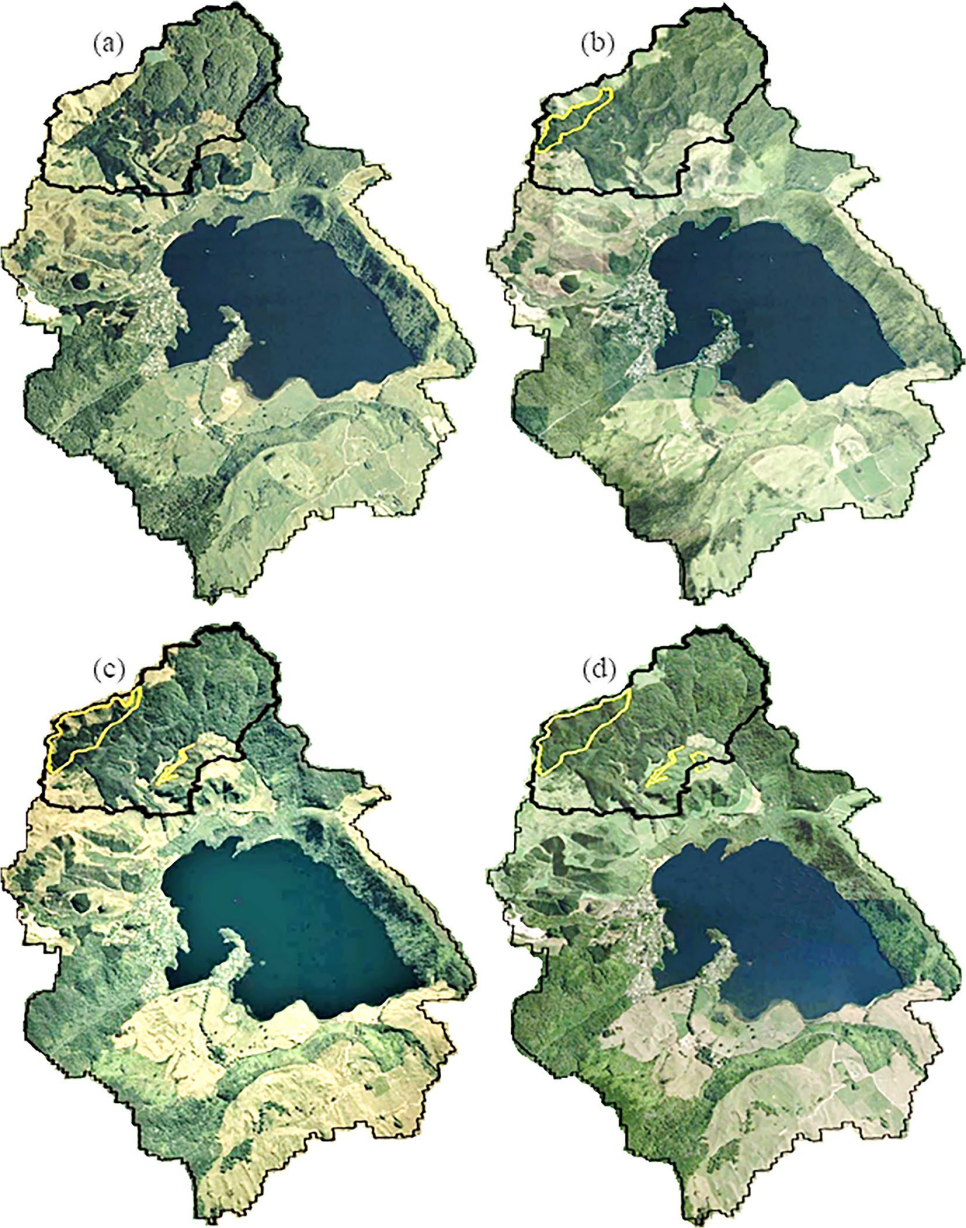
Landsat 7 (2011) and Landsat 8 (post 2013) aerial photographs of Lake Ōkareka showing post land use change in **a** 2011, **b** 2013, **c** 2015, and **d** 2022. All images are taken on the month of March (except (**d**) taken in January 2022), and yellow lines encompass areas of land use change and Millar sub-catchment is delineated with a solid black line

Landsat aerial photographs of the Lake Ōkareka catchment were used to delineate afforested areas, shown as yellow polygons in the Millar sub-catchment (Figs. 1 and 3, Table S2). Comparison of pre- and post-LUC observed streamflow (mm d^−1^) and nutrient (NH_4_-N, NO_3_-N, TN, and TP) loads (kg d^−1^) show a post-LUC decrease in mean, minimum, and maximum values across all variables, except for a small increase in minimum Q and mean and minimum TP (Table 2; see also Figs S1-S2). There was a significant decrease in post-LUC mean streamflow (*p* < 0.05), NH_4_-N (*p* < 0.01), NO_3_-N (*p* < 0.01), and TN (*p* < 0.05) but not TP (*p* < 0.05). Changes in mean values pre- and post-LUC were also assessed using a cumulative deviation *t*-statistic test (CDT) (*Ơ*, Table S3). CDT test results showed a significant post-LUC decrease (*p* < 0.05) in streamflow and loads of most of the nutrients (NH_4_-N, NO_3_-N, and TN) except for TP (*p* > 0.05).

**Table 2:** Mean observed streamflow (Q) and nutrient (NH_4_-N, NO_3_-N, TN, and TP) loads for each monitoring day at Millar sub-catchment pre (2002–2010) and post (2011–2016) land use change. NH_4_-N, NO_3_-N, TN, and TP are ammonium, nitrate, total nitrogen, and total phosphorus, respectively, and SD is the standard deviation. NS denotes not significant at a *p* value of 0.05, * is *p* < 0.05 and ** is *p* < 0.01

Variable	LUC	Mean	SD	Min.	Max.	Sample size (*n*)	*p* value
Q (mm d^−1^)	Pre	0.39	0.26	0.02	1.78	71	*
	Post	0.29	0.27	0.05	1.62	31	
NH_4_-N (kg d^−1^)	Pre	0.05	0.05	0.006	0.29	60	**
	Post	0.03	0.02	0.001	0.09	26	
NO_3_-N (kg d^−1^)	Pre	1.56	1.19	0.07	6.67	58	*
	Post	0.94	0.67	0.004	2.38	26	
TN (kg d^−1^)	Pre	2.07	1.62	0.37	10.42	55	**
	Post	1.14	0.67	0.06	2.59	26	
TP (kg d^−1^)	Pre	0.06	0.05	0.012	0.22	61	NS
	Post	0.07	0.04	0.009	0.16	26	

**Fig. 4:**
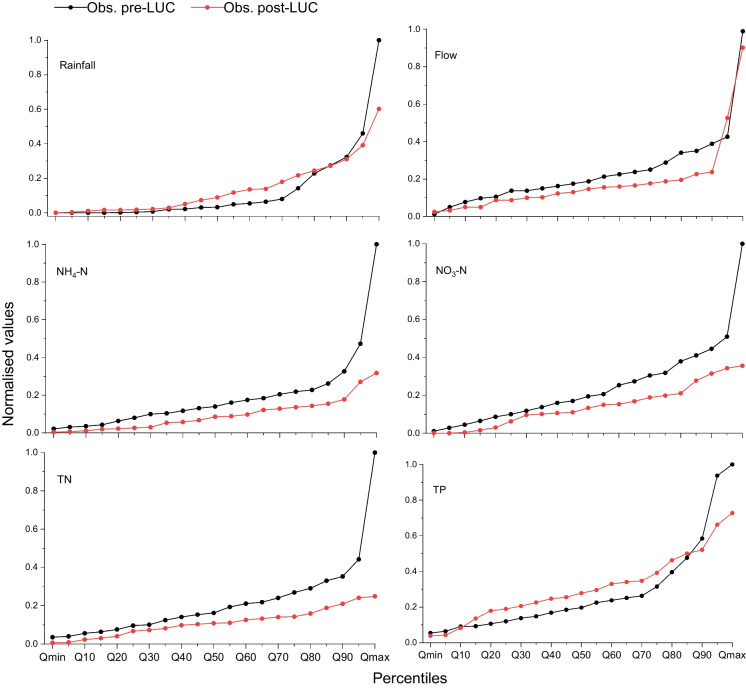
Percentile distributions of observed streamflow (mm d^−1^) and nutrient loads (kg d^−1^) of ammonium (NH_4_-N), nitrate (NO_3_-N), total nitrogen (TN), and total phosphorus (TP), and 5-day mean observed rainfall for pre-LUC and post-LUC. All variables have been normalised using maximum values of 1.80 mm (streamflow), 22.12 mm (5-day rainfall), and loads (kg d^−1^) of 0.3 (NH_4_-N), 6.7 (NO_3_-N), 10.4 (TN), and 0.22 (TP)

Non-parametric analysis was used to examine changes in streamflow and nutrient load observations pre- and post-LUC using percentile distributions. Post-LUC streamflow and water quality were mostly lower except for TP and for all percentile streamflow classes, there was a post-LUC decrease in Q, NH_4_-N, NO_3_-N, and TN (Fig. 4). TP observations had a similar post-LUC decrease for Q < 10% (i.e. the lowest 10%) and Q ≥ 90% (the highest 10%), but an increase for Q between 10 and 85%.

### SWAT model calibration and performance

We used a sensitivity analysis to provide insights into the contributions of SWAT model parameters and input data to streamflow and nutrient loads (Table 3). Sensitive flow parameters included initial Soil Conservation Services (SCS), runoff curve number for moisture condition (CN2), threshold depth of water in the shallow aquifer required for return flow (GWQMN), maximum canopy storage (CANMX), baseflow alpha factor (ALPHA_BF), aquifer percolation coefficient (RCHRG_DP), and effective hydraulic conductivity in main channel alluvium (CH_K2). Average slope steepness (HRU_SLP), a terrain parameter, varies with elevation in the catchment and affects lateral flow within the kinematic storage model in SWAT (Sloan & Moore, 1984). Lateral flow entering the stream reach is also affected by lateral flow travel time (LAT_TTIME) and slope length for lateral subsurface flow (SLSOIL).

**Table 3:** Sensitive SWAT model parameters, ranges, units, and calibrated values for streamflow (Q), total nitrogen (TN), and total phosphorus (TP) as indicated in SWAT-CUP. Note: The ‘v’ before the parameter indicates replacement of existing value by the calibrated values, the ‘a’ denotes addition of the calibrated value to the model default, and ‘r’ is for relative change, a relative change was applied by multiplying the existing value by (1 + calibrated value)

Variable	Parameter	Parameter definition	Unit	Range	Calibrated value
Q	r__CN2.mgt	SCS runoff curve number for moisture condition	–	-0.2–0.2	0.10
	v__LAT_TTIME.hru	Lateral flow travel time	day	0–180	15
	v__GWQMN.gw	Threshold depth of water in the shallow aquifer	mm H_2_O	0–5000	4500
	a__SLSOIL.hru	Slope length for lateral subsurface flow	m	0–150	48
	a__CANMX.hru	Maximum canopy storage	mm	0–100	65
	r__HRU_SLP.hru	Average slope steepness	m m^−1^	0–0.6	0.15
	v__ALPHA_BF.gw	Baseflow alpha factor	day	0–1	0.08
	v__RCHRG_DP.gw	Deep aquifer percolation fraction	–	0–1	0.09
	v__CH_K2.rte	Effective hydraulic conductivity in main channel	mm h^−1^	0–500	100
TN	v__LAT_ORGN.gw	Organic nitrogen in the base flow	mg N L^−1^	0–200	0.15
	v__ERORGN.hru	Organic N enrichment ratio	–	0–5	3.5
	v__SHALLST_N.gw	Nitrate-nitrogen concentration in the shallowaquifer	mg N L^−1^	0–1000	1.5
	v__BC3.swq	Rate constant for hydrolysis of org. N to NH_4_ in the reach	day^−1^	0.2–0.4	0.4
TP	v__LAT_ORGP.gw	Organic phosphorus in the base flow	mg P L^−1^	0–200	0.01
	v__GWSOLP.gw	Concentration of soluble phosphorus in groundwater	mg P L^−1^	0–1000	0.02
	V_ERORGP.hru	Organic P enrichment ratio	–	0–5	2

Sensitive parameters for TN included organic nitrogen in the base flow (LAT_ORGN), organic N enrichment ratio (ERORGN), nitrate-nitrogen concentration in the shallow aquifer (SHALLST_N), and reach rate constant for hydrolysis of organic nitrogen to ammonium–nitrogen (BC3). For organic phosphorus, the sensitive model parameters were baseflow (LAT_ORGP), soluble phosphorus concentration in the groundwater (GWSOLP), and organic P enrichment ratio (ERORGP). Parameters varied in the number of variables they influenced, e.g. being specific to one variable (e.g. TP), a subset of the variables (e.g. NH_4_-N and, therefore also TN), or a combination of several variables.

SWAT-CUP produced an optimised calibration for which summary statistics (*R*^2^, NSE, and PBIAS) are shown in Table 4. Based on *R*^2^ values (Moriasi et al., 2007), the goodness-of-fit of calibration and validation performance varied from very good (Q) to unsatisfactory during calibration (NO_3_-N, TN, and TP) and validation (NH_4_-N, NO_3_-N, TN, and TP), although *R*^2^ values for nutrient constituents are in the upper range of values summarised for a number of studies by Arhonditsis and Brett (2004). Additionally, according to PBIAS model performance rating criteria (Legates & McCabe, 1999), flow and nutrient variables are generally acceptably captured during calibration and validation but with slight overestimates and poor performance for the calibration of NH_4_-N and TP.

**Table 4:** Mean and median values and model simulation performance indicators using coefficient of determination (*R*^2^), Nash–Sutcliffe Efficiency (NSE), and percent bias (PBIAS) for calibration (2002–2005) and validation (2006–2010) periods for streamflow (Q), nitrate (NO_3_-N), ammonium (NH_4_-N), total nitrogen (TN), and total phosphorus (TP)

Calibration						Validation				
Variable	Q	NH_4_-N	NO_3_-N	TN	TP	Q	NH_4_-N	NO_3_-N	TN	TP
Sample size	42	38	36	33	37	29	22	22	22	24
Unit (mean/median)	mm d^−1^	kg d^−1^	kg d^−1^	kg d^−1^	kg d^−1^	mm d^−1^	kg d^−1^	kg d^−1^	kg d^−1^	kg d^−1^
Mean (obs)	0.41	0.06	1.39	1.86	0.06	0.37	0.04	1.84	2.39	0.06
Median (obs)	0.32	0.04	1.23	1.69	0.04	0.37	0.05	1.51	2.30	0.04
Mean (sim)	0.50	0.03	1.10	1.83	0.13	0.45	0.03	1.24	1.99	0.03
Median (sim)	0.41	0.02	0.96	1.60	0.04	0.40	0.02	1.18	1.86	0.01
*R*^2^	0.77^b^	0.53	0.59	0.44^c^	0.30^d^	0.73^c^	0.20	0.30	0.42^c^	0.51^c^
NSE	0.50^d^	0.20	0.36	0.40^c^	−21.63^d^	0.58^c^	−0.34	0.02	0.34^d^	0.14^d^
PBIAS (%)	−23.05^c^	53.32	21.14	1.39^a^	−123.70^d^	−21.80^c^	35.98	32.66	16.55^b^	47.52^c^

Note: Superscripts a, b, c, and d denote very good, good, satisfactory, and unsatisfactory performances, respectively (Moriasi et al., 2007; Moriasi et al., 2015). These studies do not provide a clear guideline on the performance category for NH_4_-N and NO_3_-N

**Fig. 5:**
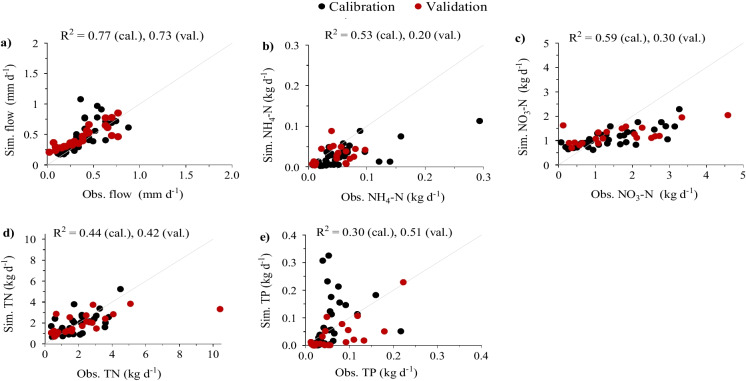
Scatterplots of observed and predicted streamflow and loads (**a**) of NH_4_-N (**b**), NO_3_-N (**c**), TN (**d**), and TP (**e**) for Millar stream for each monitoring day

Observed and simulated streamflow (mm d^−1^) and nutrient loads (kg d^−1^) were split into two time periods, pre-LUC (2002–2010) and post-LUC (post-2010). Pre-LUC observations were further split for calibration (2002–2005) and validation (2006–2010) (Table 4, Fig. 5).

**Fig. 6:**
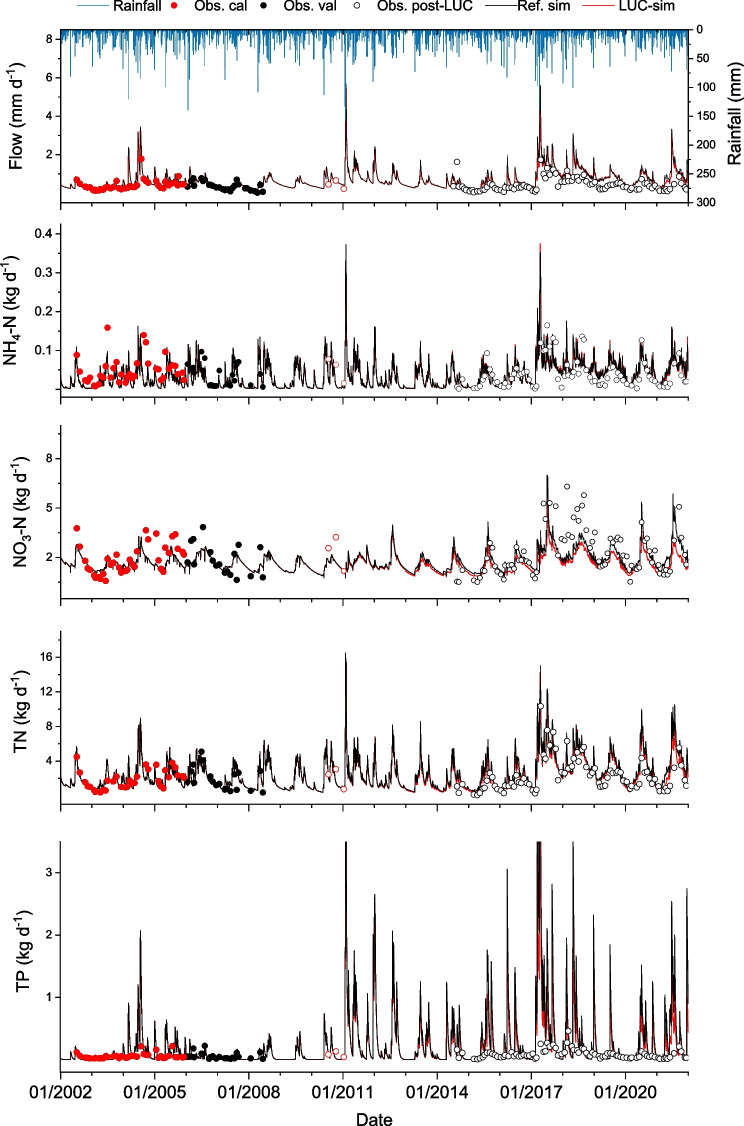
Comparison of observed and simulated streamflow (mm d^−1^) and loads (kg d^−1^) of ammonium (NH_4_-N), nitrate (NO_3_-N), total nitrogen (TN), and total phosphorus (TP) at the Millar gauging station. ‘Obs. Cal’ and ‘Obs. Val’ denote observations used for calibration (2002–2005) and validation (2006–2010) and ‘Obs. Post-LUC’ represents observation data post land use change (post-2010). Ref. sim denotes reference model simulations with a hypothetical baseline considering no LUC (S0) from 2002 to 2021 and and LUC-sim represents model predictions accounting LUC post-2010 (*S*_LUC_). Observations denoted by hollow red circles were excluded from calibration and validation

Streamflow (mm d^−1^) and nutrient load (kg d^−1^) observations and model output for Millar gauging station were split into pre-LUC (2002–2010) and post-LUC (post-2010), which included periods of calibration (2002–2005) and validation (2006–2010), Table 4 (Fig. 5). Modelled loads of NO_3_-N and TN mostly reflected seasonal variations of low flow in summer and high flow in winter, while modelled TP load also showed a similarly high level of flow dependence. Loads of NO_3_-N accounted for a high fraction of the TN (Fig. 6). The median values for model simulations were generally comparable with those of observations (Table 4). Graphical comparison of observed and predicted streamflow and NH_4_-N, NO_3_-N, TN, and TP loads for each monitoring day for Millar stream (Fig. 5) shows high similarity of modelled and observed datasets for most of the variables other than NH_4_-N, with simulated values generally exceeding the observed values.

All nutrient variables showed satisfactory performance during calibration except for TP (Moriasi et al., 2007). Visual observations of observed and predicted streamflow and water quality data for each monitoring day indicate that model simulations are satisfactory for all variables, other than TP, which could be resulted from unavailability of sediment data for calibration.

Referring to previous other studies, Moriasi et al. (2007) proposed performance categories for ‘monthly time-step’ model results and judged as satisfactory if NSE is greater than 0.50 and PBIAS < 25% for streamflow and suggested ‘appropriate adjustments’ in their criteria for annual or daily evaluation time steps. Whether this type of classification is reasonable or not needs further discussion. The four-level classification (very good, good, satisfactory, and unsatisfactory) introduced by Moriasi et al. (2007) was originally grounded in statistical analyses of diverse outcomes from hydrological model simulations. However, studies indicated that the model performance assessment criteria employed by different models lack uniformity (Lin et al., 2017).

Gupta et al. (2009) argued that in order to maximise NSE, the variability has to be underestimated which turns out to be an important feature of watersheds. McCuen et al. (2006) indicated that NSE is a single-valued measure which is overly sensitive to extreme values (Legates & McCabe, 1999) that can be influenced by several factors such as sample size, outliers, and magnitude bias and the NSE values should be accompanied by additional metrics. In this regard, we have also used *R*^2^ and PBIAS metrics to support the results, which are in the range of very good and satisfactory, respectively.

In view of other model performance metrics, our comparison of model output against measurements of flow indicated satisfactory (*R*^2^ = 0.73) and good (*R*^2^ = 0.73) values during calibration and validation, respectively (Moriasi et al., 2015). For both calibration and validation, a satisfactory PBIAS value < −25% has been achieved. From Moriasi et al. (2007), calibration and validation of nutrient variables with PBIAS < ±70% are considered satisfactory. As such, except TP during calibration, we found that all the nutrient variables ranged from satisfactory to very good performance both during calibration and validation. For analyses of other permeance metrics flow applied in our study, a NSE value of 0.5 for the daily value could be regarded as acceptable.

We also reviewed other relevant studies that have used SWAT model. A review of model performance (Moriasi et al., 2007) for daily streamflow showed NSE and PBIAS calibration values as low as −0.23 and −91.7% and validation values of −1.81 and −155.6%, respectively. Spruill et al. (2000) reported NSE values ranging from −0.04 to 0.19 for daily comparison of predicted and observed streamflow, and Vazquez-Amábile and Engel (2005) reported values ranging from −0.35 to 0.48. Van Liew et al. (2003), based on the results of five experimental watersheds in USA, reported PBIAS values from 2.9 to −91.7% for calibration and 2.7 to −155.6% for validation.

### Changes in model output pre- and post-land use change

Changes in streamflow, loads, and flow-weighted mean concentrations (FWMC) of nutrients from the SWAT model output were used to assess effects of the afforested in the Millar stream sub-catchment (Table 5). We conducted a comparison between the results generated by the SWAT model for two conditions of land use: one assuming the land use did not change post-2010 (reference simulation) and another where the existing land use changes since 2011 were taken into account (post-LUC simulation). Post-LUC, there was a 7.2% reduction in mean annual streamflow, while nutrient loads showed a reduction, from 13.1% for TN to 33.3% for TP. The change in nutrient concentrations was considerably smaller, with a reduction from 6.4% for TN to 28.1% for TP.

**Table 5:** Mean annual streamflow (Q), nutrient loads, and flow-weighted mean concentration (FWMC) from SWAT model outputs for reference simulation (ref. sim.) and land use change simulation (post-LUC) from 2011 to 2021

	Variable	Unit	Ref. sim.	Post-LUC	Absolute change	Relative change (%)	Elasticity (−)
	Q	mm yr^−1^	262.2	243.3	−18.9	−7.2	
Load	NH_4_-N	kg yr^−1^	14.0	13.7	−0.27	−2.0	0.27
	NO_3_-N	kg yr^−1^	480.2	371.4	−108.8	−22.6	3.14
	TN	kg yr^−1^	959.7	833.7	−126.0	−13.1	1.82
	TP	kg yr^−1^	122.0	81.4	−40.6	−33.3	4.61
FWMC	NH_4_-N	g m^−3^	0.015	0.014	−0.001	−6.7	0.92
	NO_3_-N	g m^−3^	0.48	0.40	−0.08	−16.6	2.30
	TN	g m^−3^	0.95	0.89	−0.06	−6.4	0.88
	TP	g m^−3^	0.12	0.09	−0.03	−28.1	3.89

The elasticity values used to test for sensitivity of nutrient loads to changes in flow ranged from 0.27 (NH_4_-N) to 4.61 (TP) loads (Table 5), i.e. a 1% change in flow produced a 4.61% change in TP. For nutrients, the FWMC elasticity values ranged from 0.88 (TN) to 3.89 (TP).

**Fig. 7:**
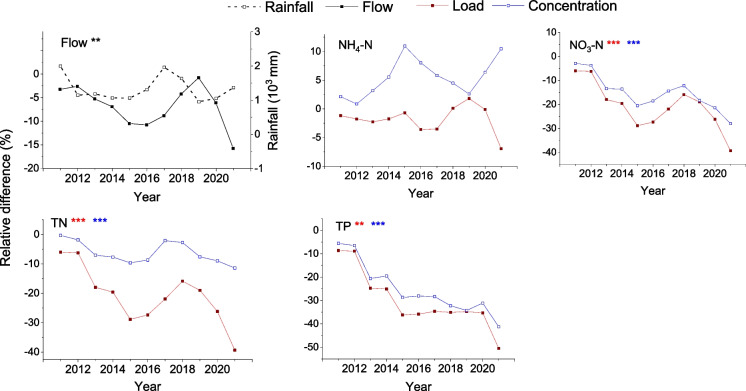
SWAT simulation output showing percent relative change in annual total streamflow (black solid lines) and loads and annual mean concentrations of ammonium (NH_4_-N), nitrate (NO_3_-N), total nitrogen (TN), and total phosphorus (TP) between reference simulation pre-LUC and post-LUC. Red and blue lines represent relative change in nutrient loads and concentrations between pre- and post-LUC, respectively (Eq. 4). Significant changes relative to the reference are given by ***(*p* < 0.001) and **(*p* < 0.01)

Percentage relative change in streamflow and nutrient loads and concentrations were compared using the SWAT model for post-LUC (*S*_LUC_) and for a hypothetical baseline (2011–2021) considering no LUC (S0). For *S*_LUC_, there was a significant reduction in annual streamflow (*p* < 0.01), and nutrient loads (*p* < 0.001, Fig. 7) and concentrations (*p* < 0.001; NO_3_-N, TN, and TP) except for NH_4_-N (*p* > 0.05).

**Fig. 8:**
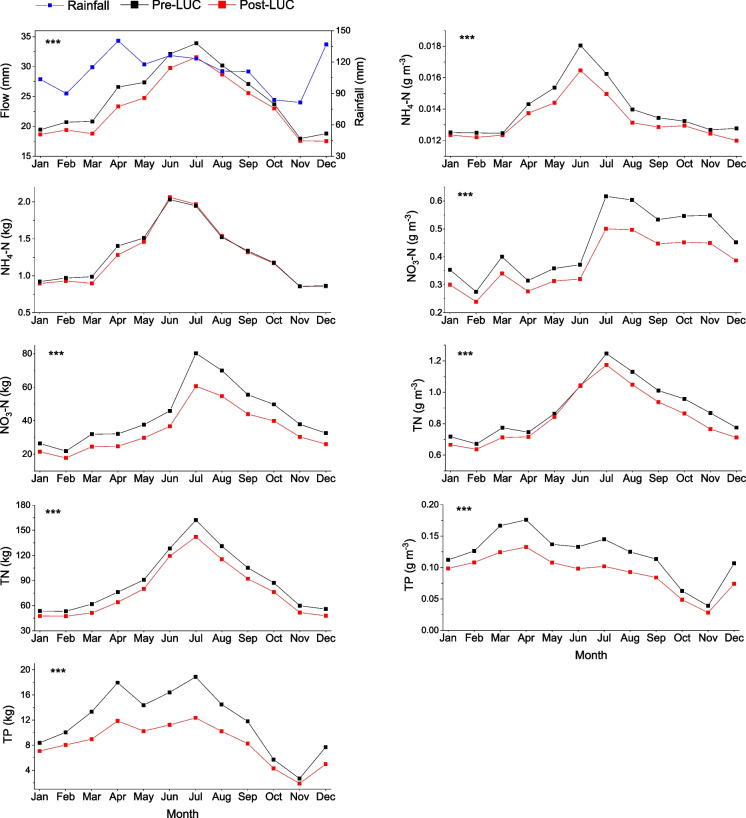
Eleven years (2011 to 2021) of monthly average SWAT model output for streamflow (mm), load (kg), and concentrations (g m^−3^) of ammonium (NH_4_-N), nitrate (NO_3_-N), total nitrogen (TN), and total phosphorus (TP) for post-LUC simulations and pre-LUC reference simulations

**Fig. 9:**
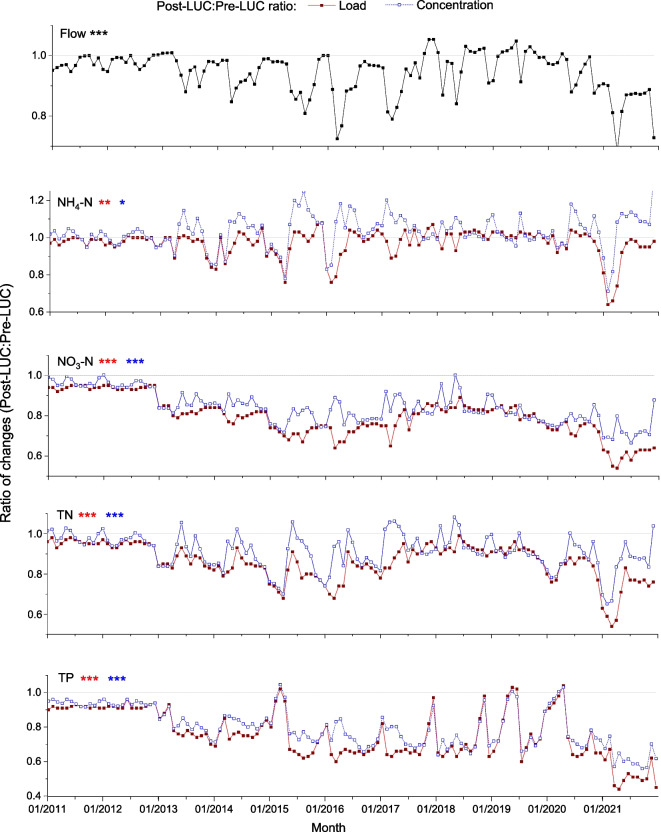
Ratio of post-LUC to reference simulation (pre-LUC) monthly mean flow and nutrient loads and concentrations from SWAT model output. Nutrients include ammonium (NH_4_-N), nitrate (NO_3_-N), total nitrogen (TN), and total phosphorus (TP). Asterisks for each variable show statistical significance of differences in loads and concentrations: * is *p* < 0.05, ** is *p* < 0.01, and *** is *p* < 0.001

Model output of monthly average streamflow and nutrient loads and concentrations from 2011 to 2021 showed marked reduction (*p* < 0.001) between the reference and the post-LUC cases, except for NH_4_-N concentrations, which showed no significant change (*p* > 0.05, Fig. 8). We also calculated the ratios of variables post- and pre-LUC from SWAT model time series of mean monthly flow and nutrient loads and concentrations. There was a statistically significant post-LUC reduction from the reference simulation for each variable in the SWAT simulations (Fig. 9).

## Discussion

Nutrient runoff from agricultural land represents a high proportion of the total nutrient yield from many catchments in New Zealand and has resulted in eutrophication of a high proportion of lakes (Abell et al., 2020a, b). As a result, water quality restoration programs involving LUC have been set up with an underlying premise that afforestation can reduce nutrient loads and address water quality problems (McDowell et al., 2020). We tested the adequacy of afforestation in a lake sub-catchment using analysis of observed data that included a non-parametric statistical analysis and a numerical modelling approach using the SWAT model. The analyses included evaluation of changes in stream discharge and nutrient concentrations before, during, and following LUC. Our hypothesis that LUC would decrease flow and nutrient loads was confirmed from results of non-parametric analyses and SWAT model outputs, with mean annual streamflow decreasing by 7.2%, nutrient loads decreasing from 2.0 to 33.3% (depending on nutrient species), and concentrations decreasing from 6.4 to 28.1% in response to LUC.

### Modelling streamflow and nutrient loads: land use change effects

Our study was conducted in a lake catchment with temporally sparse observations and discontinuous flow measurements. This situation is typical of numerous small to medium-sized catchments globally. We used non-parametric percentile distributions to assess pre-LUC and post-LUC observations and complement the analysis with SWAT model outputs, providing dual lines of evidence about the effects of afforestation and counter the situation common to many catchments where data are less than optimal. The non-parametric technique can be applied to other gauged and poorly gauged catchments where afforestation has been prescribed to meet water quality targets. The use of instantaneous flow measurements instead of daily (24-h) averages may increase model uncertainty, but such instantaneous measurements are common in monitoring programs which have budgetary and personnel constraints. The error term in our statistical comparisons of instantaneous measured flow against daily modelled values therefore includes a component of flow variability within each day, but the comparison of flow was still ‘very good’ (Moriasi et al., 2007) in our study. A study by Abell and Hamilton (2013) intensively monitored two streams for short periods in a catchment adjacent to Lake Ōkareka. Their study indicated that under baseflow, there was little flow variation within each day, but greater variability for stormflows, which varied between the streams and with recent rainfall history. Consideration should be given to continuous streamflow monitoring to better quantify contaminant loads in streams as we found that variations in flow are the dominant driver of daily load estimates compared with variations in nutrient concentrations.

Non-parametric analysis of observations and model predictions between reference simulation and post-LUC periods indicated that the model generally reproduced observed changes accurately. Model simulations showed a 7.2% reduction in mean annual streamflow corresponding to a 15% conversion of pasture to forest. Our findings are comparable to previous studies (Farley et al., 2005; Zhang et al., 2001) that have considered the effect of afforestation on changes in catchment streamflow conditions, largely due to increased evapotranspiration. A study based on analysis of measured data for over 250 catchments worldwide (Zhang et al., 2001) indicated that under similar climatic conditions, pasture crops use less water than trees, leading to greater runoff. Zhang et al. (2003) conducted a study in the Murray-Darling Basin in Australia to predict the effects of afforestation on the annual flow regime. They found that afforestation with blue gum plantations, representing 16% and 21% of sub-catchment area for two tributaries in the Goulburn Broken catchment, resulted in 8% and 14% mean annual streamflow reductions, respectively. They highlighted that the rate of reduction in mean annual streamflow initially accelerated leading up to canopy closure, as expected from changes in interception and evapotranspiration driven by leaf area, surface albedo, and rooting characteristics of plants (Vertessy, 2001).

The streamflow reduction may equilibrate as quickly as 5 years, with substantial changes within 2 to 4 years of afforestation (Farley et al., 2005). Our study showed that the rate of reduction in mean annual streamflow increased over time, from 3.2% in 2011 to 15.8% in 2021. Pine trees (*Pinus radiata*) have been used extensively for afforestation in the Lake Ōkareka catchment and more widely in New Zealand. This species has a high growth rate, high rainfall interception, and large soil moisture storage capacity due to a deep root system, as well as high evapotranspiration rates. Evaporation of intercepted precipitation is low in pastoral lands, and a 20 to 40% increase can be achieved with LUC to pine plantations (Duncan, 1995; Le Maitre, 1999), which in turn reduces streamflow and nutrient export to receiving waters (Cannell, 1999).

Trends in river water quality have been routinely analysed and reported in New Zealand (Snelder et al., 2021) and model-based assessment of the reference and current water quality status of 1031 lakes indicates a general trend of increased nutrient concentrations associated with land use intensification (Abell et al., 2020a, b). However, no direct attribution of water quality changes to land use was given. Our assessment of the water quality response to a 15% conversion of pasture to pine in a small (sub) catchment using a dual statistical and numerical model approach indicates a post-LUC reduction in nutrient loads ranging from 13.1% (TN) to 33.3% (TP). Responses of nutrient concentrations in our study were somewhat equivocal, however, with the majority of the nutrient load reduction due to a decrease in flow. The modelled decrease in annual nutrient export between the post-LUC case and a hypothetical baseline case showed load reductions ranging from 8.5% (NH_4_-N) in 2012 to 25.4% (TN) in 2021. Furthermore, as for streamflow, the annual reduction in nutrient loading increased through time. This finding is likely attributed to the initial rapid increase in forest canopy cover and the concurrent reduction in streamflow (Zhang et al., 2003).

Although direct comparison between afforestation canopy effects and nutrient load responses is difficult because of climate variability (e.g. interannual variability in rainfall), it is important to note that the TN and TP loads in post-LUC years were well below those of the first year of LUC (i.e. 2011). The initial slower response in the first year may be attributable not only to lags as trees mature but also a damping effect from a legacy of decades of pastoral land use, with soils likely to have had higher levels of inorganic nutrients. If an area remains under pine plantation (e.g. after 2–3 decades), the initial decreases in stream nutrient concentrations may begin to slow (Hughes & Quinn, 2019). Most pine plantations in New Zealand are harvested after about 25 years, however, which leads to a temporary spike (~1–2 years) in nutrient loads due to reduced evapotranspiration and abundant degradable organic matter (e.g. slash and other plant material (Hamilton, 2005). We included analysis of covariance (ANCOVA) of monthly data to assess the effects of precipitation variability on nutrient loads and to predict the relative contribution of flow and concentration on nutrient loads. Flow contributed more to NO_3_-N and TN load reductions than concentration while for NH_4_-N loads, which contribute a smaller component of TN than NO_3_-N, were nearly equally apportioned between flow and concentration.

### Land cover effects on nutrient cycling

We examined the potential impacts of land cover change on catchment hydrological and water quality responses. Changes in land use from agriculture to forest alter nutrient dynamics through changes in pools, availability, and cycling of material. Afforestation tends to generate a greater proportion of nutrients in organic form, particularly organic nitrogen, which can be relatively refractory or resistant to rapid decomposition. This characteristic of organic nitrogen in forested ecosystems can lead to slower nutrient turnover rates compared to agricultural systems. The accumulation of organic matter on the forest floor contributes to a build-up of nutrients over time, promoting nutrient retention and storage within the ecosystem.

Afforestation also alters nutrient cycling patterns within the soil and the availability oof nutrients for plants. Forests often develop a well-defined litter layer and organic-rich soil, which foster a complex and diverse soil microbial community. This soil microbial community promotes nutrient cycling and sustains the long-term fertility of forest ecosystems. A study by Łaszewski et al. (2021) revealed pronounced effects of agriculture compared with forestry on seasonal variations on NO_3_-N in a receiving stream. Wang et al. (2017) showed high potential for N_2_O emissions, denitrification activity, and denitrifier abundances in rural farmland soils compared turfgrass soils. Tong et al. (2019) observed substantial soil organic carbon and TN losses when woodland and grassland were converted to cultivated land types. Similarly, Kooch and Noghre (2020) demonstrated due to variations in soil organic matter while in the tropics, Kuma et al. (2022) showed high rates of nutrient loss in agricultural catchments. These findings collectively reveal that land cover plays a crucial role in nutrient cycling but, as shown in our study, delineation to individual subcatchment or stream scale may be required to identify causal relationships.

### Catchment management implications

The New Zealand government is implementing freshwater restoration programs, supported by national policies and regional implementation of action plans. The regional council action plan target to reduce the potential for degradation of water quality of Lake Ōkareka was a 0.9 t yr^−1^ reduction in TN and 0.05 t yr^−1^ reduction in TP, estimated to be achieved through a 200 ha LUC from pasture to trees (EBoP, 2004). We conducted this research to assess the validity of land use change in Lake Ōkareka according to nutrient load targets in an action plan but also to assess resilience to greater variability of rainfall, which may be expected with climate change (Millar et al., 2007). The conversion of low intensity pastoral land use to forest in a sub-catchment (Millar) representing 15% of the total lake catchment area, with reduction in nutrient loads of 13.1% (TN) to 33.3% (TP) in the subcatchment, appears to validate the approach taken for Lake Ōkareka. Several other lakes in the region are subject to action plans, with reductions in nutrient export vital to enhance lake water quality (Monaghan et al., 2021).

Our study provides policy support and scientific evidence for a model catchment, being suitable for others where similar catchment actions are planned at national, regional, and local levels. Nationally, the Rotorua Regional Council is required to demonstrate that it complies with the National Policy Statement for Freshwater Management (Ministry for the Environment, 2020) that requires regional councils to set nutrient concentrations and exceedance criteria so that diffuse nutrient pollution does not adversely impact receiving waters. Our findings can support the regional council in assessments if further conversion of pasture to trees is adequate to avoid eutrophication of the downstream receiving waters of Lake Ōkareka and thereby serve as a model for other catchments in New Zealand where water quality of receiving waters is impaired.

At regional level, our study can help to inform policy makers in the implementation of the Regional Water and Land Plan (Rule 11) which seeks to address the export (diffuse discharge) of nutrients from land use activities (Foster & Kivell, 2009). In addition, this research provides important insights on the extent of land use change required and the latency of catchment response to land use change. This information is especially pertinent where large land use change investments are being made to support water quality goals. Water quality constituents have different responses to rainfall, which drives much of the short-term variability in concentrations. The particulate P component of TP is closely related to surface runoff and sediment erosion events while dissolved N varies mostly with changes in nitrate due to changes in groundwater delivery during storm events (Abell et al., 2013). Our study had some limitations, one of which was inadequate observed data to support modelling of suspended sediments. A substantial component of the phosphorus load could be expected to be in particulate form although there can be interactions with dissolved phosphorus through adsorption and desorption, depending on phosphorus isotherm kinetics of the sediment (Peryer-Fursdon et al., 2015). Future monitoring of the stream with continuous streamflow and nutrient and suspended sediment measurements would help to separate short-term changes (e.g. from storm events) from long-term changes due to effects of land use change and improve quantification of loads of particulate and dissolved phosphorus. Measured data and calibration of the model on paired subcatchments (e.g. control and test cases) would also allow identification of LUC or climate effects when one catchment is subjected to LUC treatment while the other remains as a control. The paired catchment should be located in close proximity to ensure similar characteristics of climate, soils, area, slope, aspect, and vegetation.

## Conclusions

Conversion of pastoral areas to forestry is purported to improve water quality of receiving waters but few studies have used different approaches to quantify the water quality changes. Validation is often complicated by sparse monitoring data that makes it difficult to detect changes due to mitigation actions. This situation is common for numerous small to medium-sized catchments globally and complicates the delivery of evidence-based science to inform policy for land use change. We used a dual approach involving statistical analysis of discrete observations and dynamic simulation modelling to provide independent lines of evidence to assess the effect of land use change on water quantity and quality. This approach is valuable for assessing whether actions designed to meet nutrient load targets for a catchment will be attained and over what time frame, including the lag times for land use change to impact hydrology and water quality. The numerical modelling approach can offer insights into how a future climate might impact the targets and ultimately the water quality of receiving waters. To assess how water quality has varied during different flow regimes and to understand future trends under different climatic conditions, further research and policy directions would be needed to have a network of stream gauges with continuous flow and water quality measurements representative of the spatiotemporal variations. In addition, TP is closely related to surface runoff and sediment erosion events. However, in sparsely monitored catchments where there is not sufficient sediment data, this connection is not adequately represented. Therefore, future research and monitoring efforts should focus on obtaining continuous sediment data to improve our understanding and modelling of catchment TP dynamics.

### Supplementary information

ESM 1 (DOCX 225 kb)

## Data Availability

Climate datasets that support the findings of this study are openly available in the NIWA National climate database at cliflo.niwa.co.nz/. Streamflow data is available from Bay of Plenty Regional Council Environmental Data Portal at https://envdata.boprc.govt.nz/data and Water quality data was obtained from Bay of Plenty Regional Council. Digital soil data is openly available at smap.landcareresearch.co.nz/home and land use data can be accessed from www.lcdb.scinfo.org.nz/about-lcdb).

## Abbreviations

ANCOVA: analysis of covariance
BoPRC: Bay of Plenty Regional Council
CDT: cumulative deviation
DEM: digital elevation model
FWMC: flow-weighted mean concentrations
HRUs: Hydrologic Response Units
LCDB-2: Land Cover Database v2
LUC: land use change
LUP: land use update
MFA: Ministry of the Environment
MINP: mineral phosphorus
NSE: Nash–Sutcliffe Efficiency
NO_3_-N: nitrate
NH_4_-N: ammonium
NIWA: National Institute of Water & Atmospheric Research
NPS: non-point source
NPS-FM: National Policy Statement for Freshwater Management
NZLRI: New Zealand Land Resource Inventory
ORGN: organic nitrogen
ORGP: organic phosphorus
P: phosphorus
Post-LUC: post-land use change
Pre-LUC: pre-land use change
Q: streamflow
*R*^2^: coefficient of determination
RDC: Rotorua District Council
SD: standard deviation
SWAT: Soil and Water Assessment Tool
SWAT-CUP: SWAT-Calibration and Uncertainty Program
SPARROW: SPAtially Referenced Regression On Watershed
SUFI-2: Sequential Uncertainty Fitting 2
TN: total nitrogen
TP: total phosphorus
