# Supplementary material for: Response of streamflow and nutrient loads in a small temperate catchment subject to land use change
Source: Environ Monit Assess. 2023 Nov 6;195(12):1418. doi: 10.1007/s10661-023-11828-z (PMC10628004; doi:10.1007/s10661-023-11828-z)
Supplement: Supplementary file 1 — (DOCX 225 kb) [file 10661_2023_11828_MOESM1_ESM.docx]

## Response of streamflow and nutrient loads in a small temperate catchment to land use change

Gebiaw T. Ayele ^a^, Bofu Yu ^a^, Andy Bruere ^b^, David P. Hamilton ^a, *^

^a^ Australian Rivers Institute and School of Engineering, Griffith University, Nathan, Queensland 4111, Australia

^b^ Bay of Plenty Regional Council, Rotorua, New Zealand

* Corresponding author: David P. Hamilton, Email: [david.p.hamilton@griffith.edu.au](mailto:david.p.hamilton@griffith.edu.au)

**Supporting information**

**Table S1** Classical objective functions, characteristics, function category, equation, and performance classification ratings for flow in a monthly time step.

| **Model performance ratings** | | | |  |  |  |
| --- | --- | --- | --- | --- | --- | --- |
| Objective Function |  | Statistic equation | | Value range | Performance Classification | References |
| NSE |  | *NSE = 1 −* $\left[ \frac{\sum_{i=1}^{n} {(Q}_{m}-{Q_{s})}^{2}}{\sum_{i=1}^{n} {(Q}_{m}-{\bar{Q}_{m})}^{2}} \right]$ | | 0.75 < NSE ≤ 1 | Very good | (Moriasi et al., 2007) |
|  |  |  | | 0.65 < NSE ≤ 0.75 | Good |  |
|  |  |  | | 0.5 < NSE ≤ 0.65 | Satisfactory |  |
|  |  |  | | NSE ≤ 0.5 | Unsatisfactory |  |
| R^2^ |  | R^2^ *=* $\left[ \frac{\sum_{i=1}^{n} \left[ \left( Q_{m, i}-\bar{Q}_{m} \right)\left( Q_{s, i}-\bar{Q}_{s} \right) \right]}{\sqrt{\sum_{i=1}^{n} \left( Q_{m, i}-\bar{Q}_{m} \right)^{2}}\sqrt{\sum_{i=1}^{n} \left( Q_{s, i}-\bar{Q}_{s} \right)^{2}}} \right]^{2}$ | | > 0.85  0.75 < R^2^ < 0.85  0.60 < R^2^ < 0.75  R^2^ < 0.60 | Very good  Good  Satisfactory  Unsatisfactory | (Moriasi et al., 2015) |
| +PBIAS |  | *PBIAS=* $\left[ \frac{\sum_{i=1}^{n} \left( Q_{m}-Q_{s} \right)}{\sum_{i=1}^{n} \left( Q_{m} \right)} \right]*100\%$ | | PBIAS < ±10  ±10 ≤ PBIAS < ±15  ±15 ≤ PBIAS < ±25  PBIAS ≥ ±25 | Very good  Good  Satisfactory Unsatisfactory | (Moriasi et al., 2007) |

Note: n is the number of pairs of measured and simulated variables, i is the time series of the measured and simulated pairs; *s* and *m* denotes simulated and measured datasets, respectively, and the bar stands for average. R^2^ is the coefficient of determination; NSE is the Nash–Sutcliffe efficiency; and PBIAS is percent bias.

**Table S2** Land use (%) in the Millar stream catchment (area = 384 ha).

| Year | Forest ^a^ | | Shrub and grazing lands | |
| --- | --- | --- | --- | --- |
|  | Native | Pine | Rangeland | Pasture |
| 2003 | 37 | 13 | 7 | 43 |
| 2011 | 37 | 17 | 7 | 39 |
| 2013 | 37 | 27 | 7 | 30 |
| 2015 | 37 | 28 | 7 | 28 |
| 2019 | 37 | 28 | 7 | 28 |
| 2021 | 48 | 28 | 4 | 20 |
| 2022 | 48 | 28 | 4 | 20 |

^a^ Pine (*Pinus radiata*) is an exotic plantation species and native forest includes species that are endemic to New Zealand.

The observed difference between pre- and post-LUC streamflow and nutrient load monitoring data is indicated by the result of a cumulative deviation (CDT) t-statistic tests. CDT t-test statistics (Ơ) of each variable for the length of observation (n), table of critical values of Q/√n at significance level (p) is indicated in Table S3. Comparison of pre- and post-LUC observations for flow (p < 0.05), NH_4_-N (p < 0.01), NO_3_-N (p < 0.05), and TN (p < 0.01) indicates significantly higher mean post-LUC reduction in flow, NH_4_-N, NO_3_-N, and TN (P < 0.05). A smaller p value indicates that the null hypothesis is rejected and there is a change between means of pre- and post-LUC observations. On the other hand, observed difference between pre-LUC and post-LUC TP sample means was not significantly different at p < 0.05.

**Table S3** File summaries of results from cumulative deviations (CDT) statistical testing for streamflow (Q), ammonium (NH_4_-N), nitrate (NO_3_-N), total nitrogen (TN), and total phosphorus (TP) between pre- and post-LUC periods. NS denotes not significant at a p value of 0.05, * is p <0.05 and ** is p < 0.01.

| Variable | Sample size (n) | Test statistic (Ơ) | Critical values (Ơ/√n) |  | p value |
| --- | --- | --- | --- | --- | --- |
| Q | 102 | 14.3 | 1.42 |  | * |
| NH_4_-N | 86 | 14.7 | 1.58 |  | ** |
| NO_3_-N | 84 | 12.5 | 1.37 |  | * |
| TN | 81 | 13.8 | 1.54 |  | ** |
| TP | 87 | 9.1 | 0.97 |  | NS |

**
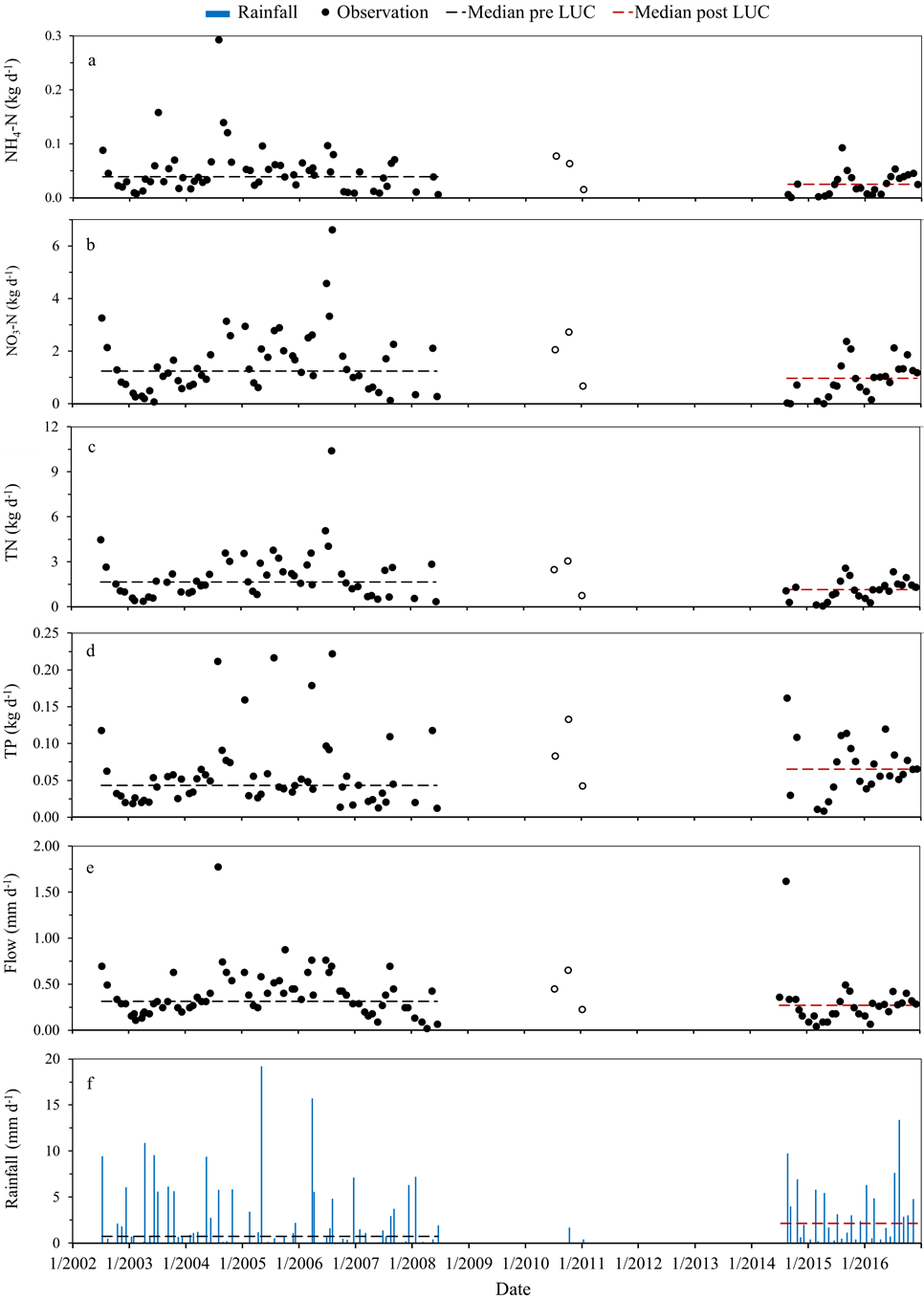
**

F**ig. S1** (a-d) observed nutrient loads, (e) streamflow, and (f) 5-day mean rainfall during pre- and post land use change periods. Black/red-dashed lines represent pre- and post-LUC medians for flow and nutrient loads and the average 5-day rainfall. Observations during 2009-2014 (hollow circles) were not accounted as either pre-or post-LUC data during calculation of changes in flow, nutrient load, and 5-day rainfall.

**Fig. S2** A box plot of pre-and post-LUC observations. The boxes represent the 25 and 75^th^ percentiles (interquartile range, IQR), the whiskers represent the 10 and 90^th^ percentiles, and the rectangles inside each box denotes the mean

**References**

Moriasi, D. N., Arnold, J. G., Van Liew, M. W., Bingner, R. L., Harmel, R. D., & Veith, T. L. (2007). Model evaluation guidelines for systematic quantification of accuracy in watershed simulations. *Transactions of the ASABE, 50*(3), 885-900, <https://doi.org/10.13031/2013.23153>.

Moriasi, D.N., Gitau, M.W., Pai, N., and Daggupati, P. (2015). Hydrologic and water quality models: Performance measures and evaluation criteria. *Transactions of the ASABE* 58(6)**,** 1763-1785. doi: https://doi.org/10.13031/trans.58.10715.
